# A Concept of Bayesian Regulation in Fisheries Management

**DOI:** 10.1371/journal.pone.0111614

**Published:** 2014-11-03

**Authors:** Noél Michael André Holmgren, Niclas Norrström, Robert Aps, Sakari Kuikka

**Affiliations:** 1 Systems Biology Research Centre, School of Bioscience, University of Skövde, Skövde, Sweden; 2 University of Tartu, Estonian Marine Institute, Tallinn, Estonia; 3 Fisheries and Environmental Management Group, Department of Environmental Sciences, University of Helsinki, Helsinki, Finland; North Carolina State University, United States of America

## Abstract

Stochastic variability of biological processes and uncertainty of stock properties compel fisheries managers to look for tools to improve control over the stock. Inspired by animals exploiting hidden prey, we have taken a biomimetic approach combining catch and effort in a concept of Bayesian regulation (BR). The BR provides a real-time Bayesian stock estimate, and can operate without separate stock assessment. We compared the performance of BR with catch-only regulation (CR), alternatively operating with *N*-target (the stock size giving maximum sustainable yield, MSY) and *F*-target (the fishing mortality giving MSY) on a stock model of Baltic Sea herring. *N*-targeted BR gave 3% higher yields than *F*-targeted BR and CR, and 7% higher yields than *N*-targeted CR. The BRs reduced coefficient of variance (CV) in fishing mortality compared to CR by 99.6% (from 25.2 to 0.1) when operated with *F*-target, and by about 80% (from 158.4 to 68.4/70.1 depending on how the prior is set) in stock size when operated with *N*-target. Even though *F*-targeted fishery reduced CV in pre-harvest stock size by 19–22%, it increased the dominant period length of population fluctuations from 20 to 60–80 years. In contrast, *N*-targeted BR made the periodic variation more similar to white noise. We discuss the conditions when BRs can be suitable tools to achieve sustainable yields while minimizing undesirable fluctuations in stock size or fishing effort.

## Introduction

Fisheries managers are challenged with two widely permeated properties of their study system, uncertainty [1]–[3] and variability [4]–[6]. These can confound each other, e.g. imprecise spawning stock size estimates can generate apparent variability in the stock − recruitment relationship [7]. Reduced inter-annual variability in effort has socio-economic benefits with yields matching the capacity of the processing industry [8], a more stable job market, and fewer years with over-dimensioned fleets [9]. Often there is a trade-off between maximizing yield and stabilizing yield and fishing effort that makes management objectives ambiguous [10]–[12]. There are also concerns that fishing increases the temporal variability of harvested stocks, [4], [13]–[15].

The problem with temporal variability in fisheries has led to an increased interest in the performance of alternative harvest control rules [16]–[18]. Stephenson et al. [19] argue that the total allowable catch (TAC) could be used to prevent unsustainable use, but is insufficient to control spatial and temporal variability, and cannot be used to achieve socioeconomic objectives. In a reflection over proposed alternative harvest controls, May et al. [20] conclude that further mathematical refinement is probably not as important as developing “robustly self-correcting strategies that can operate with only fuzzy knowledge about stock levels and recruitment curves”. If our belief of the stock size is a probability function (in contrast to a point estimate), Bayes’ theorem postulates that harvesting information can be used to calculate a conditional probability function [21]. Here we propose Bayesian regulation (BR) that uses catch- and effort data from the ongoing fisheries to make real-time estimates of stock size and fishing mortality. The years can be linked by using the posterior distribution as a prior in the sequential year, and hence exploitation is combined with stock size assessment. Real-time assessment of BR would be advantageous to management routines relying on forecasts of stock abundances. Given that most commercial fisheries with CR-based control of biomass and fishing mortality (e.g. TAC) routinely monitor effort and catch, this additional information is surprisingly poorly utilized during the CR fishing season. We suggest a methodology where this information can be used for updating population size estimates to facilitate in-season management decisions.

The BR is derived from Bayesian foraging theory in behavioral ecology, which describes a giving-up rule for patch foraging animals [22]–[24]. The rule is a relaxation of the full information assumption of the marginal value theorem [25], with search time and number of prey caught as information variables. It applies to patches in which the prey are hidden, such as a woodpecker feeding on pupae under bark [26], [27]. Given some prior information available to the forager, e.g. experience from foraging in patches of its territory, a Bayesian posterior distribution can represent the forager’s continuously changing belief about the prey density in the current patch [28]. The animal maximizing its intake rate would thus leave the patch for a new one when the anticipated intake rate in the current patch drops below the one expected from patches on average in the territory. The expected number of remaining prey in a patch can be expressed with a fairly simple equation derived by Iwasa et al. [29], but the decision to leave should include a discounting of the value of further bits of information [30].

For fisheries applications, we have modified Iwasa’s equation for Bayesian prey density estimation for recurrent exploitation of one population. Both are cases of Bayesian update of mean and variance of a population size estimate. In the original scenario, the exploiter is informed by the mean and variance of several exploited patches within its foraging area. In the fishery scenario, the exploiter (manager) is informed by previous exploitations and surveys in the same area, followed by quantitative assessment and stochastic forecast simulations. We compare CR with BR, and show how they perform in relation to management objectives and targets, by simulating fishing on a model of the main basin Baltic Sea herring (Appendix S2) [31]. We present levels and temporal variability in yield, fishing mortality, stock abundance, spawning stock biomass (SSB), and finally how frequently it surpasses the MSY related reference point *B*_trigger_ [32].

## Methods

We look at three levels of decision-making in fisheries management as they are described in the common fisheries policy of the Council of the European Union [33]: (i) The *management objective*, which is the ultimate goal of fisheries management. The objective can be simple or more complex, for example weighing incompatible goals [34], [35]. We have chosen the maximum sustainable yield (MSY) because it is the current objective of fisheries management in the EU [36]. (ii) The *target* of exploitation, which should be given in a quantitative unit that relates to the management objective. We compare the efficiency of two targets: fishing mortality (*F*_MSY_) and post-harvest stock size (*Ń*_MSY_). (iii) The *harvest rules,* which “[lay] down the manner in which annual catch and/or fishing effort limits are to be calculated and provide for other specific management measures, taking account also of the effect on other species” [33]. On this level we choose to use other terms. We use the term *regulation type* to denote the use of catch and/or effort regulation, whereas the term *harvest control rule* (HCR) is used for the combined objective, target and regulation type.

The harvest control rules under development by the International Council for the Exploration of the Sea (ICES) involve a fishing mortality target (F*) and the spawning stock biomass reference point *B*_trigger_ [32], [37]. If the SSB goes below *B*_trigger_ the fishing mortality is reduced below target, a situation that should warrant a reinvestigation of the stock condition and the harvest control [32]. Based on a stock assessment and a short-term forecast, the fishing mortality target is recalculated as a TAC, which is how the recommendation is presented to the European Commission [38]. ICES harvest control rules use precautionary reference points below which fishing mortality is reduced [32], something we do not use in our simulations. We have focused on the principle differences between CR and BR, e.g. the elaborate data collection and assessment procedure is simplified by using the actual size of the simulated stock with a random error.

### Harvest control

Altogether we compared eight different harvest controls achieved by the combinations of two exploitation targets, two regulation types and two stock assessment modes (Table 1). The Bayesian regulation *BR*(*γ, f*|*µ, ν*) is a function of catch (*γ*) and effort (*f*) given the prior information of the estimated stock size before harvest, *µ*, and the uncertainty of that estimate given as variance, *ν*. The Bayesian estimate of the number of remaining individuals (*r*) in a harvested stock is:
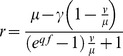
(1)where *q*, the catchability, is defined as the fraction of the population captured by one unit of fishing effort and is the scalar between catch per unit effort indices and average population abundance, hence 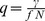. When fishing is closed by fulfillment of the target condition, *f* = *f* *, then the fishing mortality *F = q f* *. This is a generalization of Iwasa’s [29] Bayesian estimates of remaining prey population, see Appendix S1 for details on how Equation (1) is derived from some specific probability distributions. With a known prior probability distribution and a given catch and effort, a posterior distribution can be calculated with *r* being the mean. It is very unlikely that the prior mean is equal to the actual population size. The posterior mean will approach the actual value with accumulating catch and effort, but there will always be a bias towards the prior [39]. For large deviations of the prior mean in relation to the real population size, it may take more than one year to track the population size more closely. Hence, there can be temporal correlations in estimate biases. This depends on the random perturbations displacing the population size from the prior mean and the harvesting information making the posterior mean approach the real value.

**Table 1:** Characteristics and settings for the evaluation of eight different harvest controls: CR being a simple catch-only regulation; BR is the proposed regulation combining catch and effort.

Harvest	Target	Supervision	Prior	Prior
control			*µ*	*v*
CR_F_^s^	*F*_MSY_	Supervised	*N*+*ε*	0
CR_F_^u^	*F*_MSY_	Unsupervised	*N*_MSY_	0
CR_N_^s^	*N*’_MSY_	Supervised	*N*+*ε*	0
CR_N_^u^	*N*’_MSY_	Unsupervised	*N*_MSY_	0
BR_F_^s^	*F*_MSY_	Supervised	*N*+*ε*	var(*N*_MSY_)
BR_F_^u^	*F*_MSY_	Unsupervised	*N*_MSY_	var(*N*_MSY_)
BR_N_^s^	*N*’_MSY_	Supervised	*N*+*ε*	var(*N*_MSY_)
BR_N_^u^	*N*’_MSY_	Unsupervised	*N*_MSY_	var(*N*_MSY_)

The subscript denotes type of target and the superscript the existence of supervision. The target column denotes the targets used: *F*_MSY_ is the fishing mortality giving highest yield in the MSY-analysis of the SOM, and *N*’_MSY_ is the corresponding post-harvest stock size. The management is either supervised with a separate assessment or unsupervised (see methods). For unsupervised management we use the mean, *µ*, and the variance, *v*, of the pre-harvest stock size from MSY-analyses: *N*_MSY_. In supervised management *µ* is instead the actual pre-harvest stock size, *N*, with an added randomly generated error term *ε* drawn from the normal distribution, (mean = 0, var = var(*N*_MSY_) where var(*N*_MSY_) is taken from an MSY-analysis).

**Figure 1:**
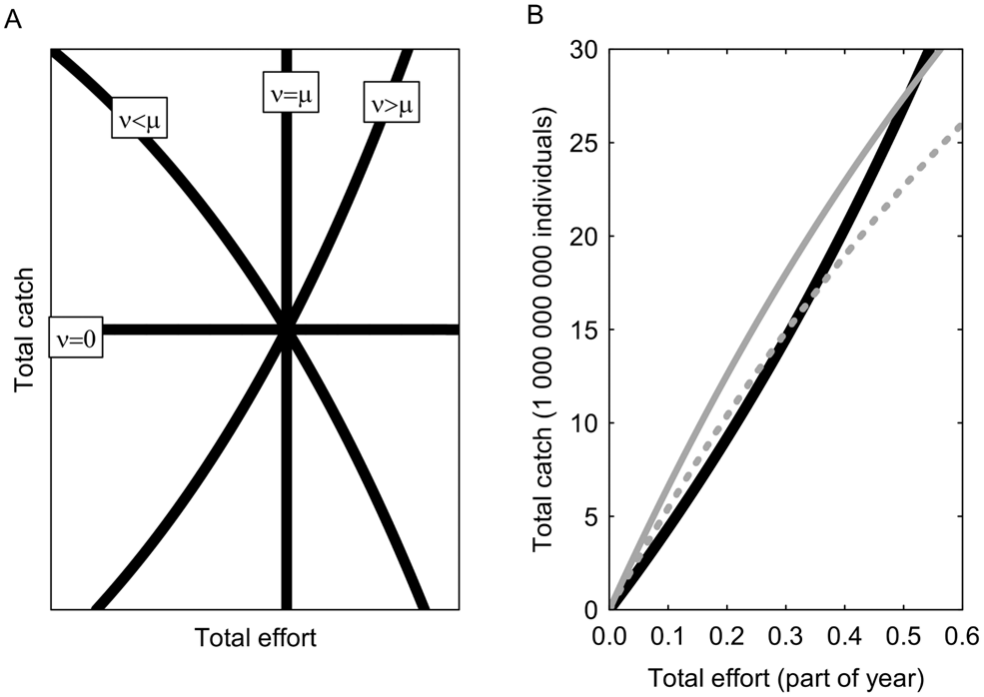
(A) Bayesian regulation curves indicating the combined total catch (y-axis) and total effort (x-axis) when fishing should be terminated for the season. The curves are given by equation 1 when *r* is set to the target number of fish, and solved for total catch. Hence, the curves show when the fishery target is achieved in catch-effort space, given by the ratio of variance (*v*) to mean stock size (*µ*). There are four qualitative cases of the *v-µ* ratio: when the ratio is zero, the fishery is regulated by catch only because there is no uncertainty around the mean. When the ratio equals 1, regulation is by effort only. Apart from these special cases, the fishery should be regulated by both catch and effort. If the variance is lower than the mean, the posterior mean will decrease with accumulating catches and effort. If the variance is higher than the mean, the posterior mean will increase with increasing catches but decrease with effort. These effects can be deduced from analyzing equation 1. (B) Here the solid curve denotes the Bayesian regulation when *v*>*µ*, which is determined prior to the fishing season (see Methods). The grey curves are idealized trajectories of how cumulative catch and effort develops from the origin during the fishing season. When the trajectories cross the regulation curve, the Bayesian posterior is on target, and the fishery should be closed for the season. The hatched grey line indicates when the initial stock size is at the prior mean (*µ*), the solid grey line when the stock size is at the mean plus one standard deviation (see *F*_MSY_ -analysis). The BR thus allows larger catches when the stock size has been underestimated, and vice versa.



When Equation (1) is solved for catch as a function of *f*, and *r* is given a target value, it can be visualized as harvest control curves (Fig. 1A). These curves can be calculated prior to the fishing season and define the combination of total catch and total effort at which the Bayesian information indicates that the fishery has reached its target. The real-time accumulation of catch and effort during the fishing season is responsive to over- and under-estimation of stock size and will develop different trajectories. The intersection of the real-time accumulation of catch and effort with the BR-curve gives the total catches when an initial over- or under-estimation is compensated for (Fig. 1B). Note that if we have no uncertainty in our prior estimate, i.e. *v* = 0, Equation (1) simplifies to *r = µ*−*γ*, in other words the remaining individuals in the population is our prior estimate minus the catch size. This means that catch-only regulation is a special case of BR (Fig. 1A). To be used in fisheries, Equation (1) needs to account for the simultaneous removal by predators (*M*), which is done by introducing two scaling parameters *α* and *β*:
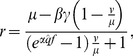
(2)where parameter 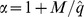 simply scales up the effort with the total mortality in proportion to 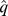. The notation 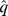 denotes the assumed value of the catchability. As default 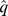 is constant and unbiased, but we make separate runs to explore the impact of biases and stochastic errors in 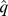. Catchability is difficult to determine accurately and can be affected by aggregation behavior of fish and technical enhancement of fishing gear [40]. Parameter *β* scales the catch γ to the total number of casualties from fishing and natural mortality:

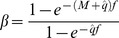
(3)Note that *β* depends on effort such that Equation (2) can work as a real-time estimator of the population size. The Bayesian estimator is used with the different targets (Table 1). When the target is formulated as a post-harvest population size (*N*’_MSY_), harvesting should stop when the Bayesian estimate of remaining stock size (*r*) equals target *N*’_MSY_:
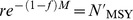
(4)


The target *N*’_MSY_ denotes the stock size at the end of the year, we therefore need to take into account the removal of individuals due to natural mortality for the remaining part of the year after fishing has ended, 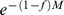. When our target is defined as fishing mortality, *F*_MSY_, harvesting is aborted when.
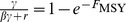
(5)


Note that the prior *µ* cannot be used in the denominator, because the estimated size of the pre-harvest population changes as we receive information from catch and effort. When the conditions in Equation (4), or (5) are fulfilled, we extract the fishing effort (f*) from Equation (2). Equations (2), (4) and (5) together define the control rules both for CR and BR.

### Assessment and priors

Estimates of the pre-harvest stock size, the prior *µ*, are produced in two ways: by supervised assessment and unsupervised assessment (Table 1). Supervised assessment represents the current annually revised assessment practiced by ICES and other stock assessors. In this case, *µ* is calculated by adding an assessment error to the actual pre-harvest stock size, *N*. The error is a random value drawn from a normal distribution with mean* = *0, and variance_v_ which is equal to the variance of the pre-harvest stock size in the *F*_MSY_-analysis (see below). The coefficient of variance in the *F*_MSY_-analysis is 21%. This represents an ideal assessment where the error solely stems from the yearly variation of the population when fished at *F*_MSY_. The unsupervised assessment uses *µ* equal to the average, and *v* the variance of the pre-harvest stock size from the *F*_MSY_ -analysis (i.e. when fishing at *F*_MSY_; see below). The rationale of using a constant *µ* is that the Bayesian information of stock size when a fishery has been closed in the foregoing year indicates *N*’_MSY_ at the end of the year when using *N*-target (Equation 4). Similarly for an *F*-targeted fishery, fishing is closed every year when at estimated *F*_MSY_, which is associated with the mean *N*’ = *N*’_MSY_. The variance is used for the BR, whereas *v = *0 for the CR.

### The stochastic operating model

We have used a stock model of the herring population in the main basin of the Baltic Sea (ICES catch area subdivisions 25–27, 28.2, 29 and 32) for harvest control evaluation. The model is a stochastic operating model (SOM) parameterized from statistical analyses of ICES catch data and outputs from ICES XSA runs (Appendix S2).

### MSY analysis

We performed an MSY-analysis on the SOM by stepping the fishing mortality in steps of 0.01, and for each *F*-value simulating 40,000 years and rejecting the first 500 to minimize the effects of initiation values. In contrast to simulated management, this algorithm executes perfectly-controlled constant fishing mortality. The relationship between yield and fishing mortality was used to identify the *F*_MSY_ and its associated average post-harvest stock size. These were used as targets for *F*-targeted and *N*-targeted management, respectively. The average pre-harvest stock size, given fishing mortality *F*_MSY_, was used as the prior (*µ*) in the unsupervised simulations, and the variance in the pre-harvest stock size was used as a measure of the uncertainty (*v*) of *µ* in the BR. The lower 2.5% percentile of the associated SSB was used as the *B*_trigger_. Targets, priors and assessment modes were used in defining the eight different HCRs, all aiming at achieving MSY (Table 1).

### Evaluation of harvest control rules

The eight HCRs were applied to Monte Carlo simulations of the SOM. A simulation started with a number of years of controlled fishing with *F*_MSY_ to get away from the initial population size and age-structure. After the initial period, data was collected for a number of years of applied HCR. We ran shorter time series with an initiation period of 1,000 years and 100 years of applied HCR. The time period of 100 years reflects a reasonably long time-horizon for management. We also ran longer series with an initiation period of 100 years and 20,000 years of applied HCR, in order to establish more accurate estimates of mean yields. We collected annual data on yield, fishing mortality, pre-harvest and post-harvest population size, and SSB. Cod SSB and year-specific growth were kept at a mean level (*C_y_* = 100,000 tons, *k_y_* = 0) during the simulations with the addition of random noise, the size of which was extracted from historical data after removing long-term changes [31]. Technically, fishing on the SOM is performed by applying the effort, f*, from the solution of the quitting rules in Equation (4) and (5). f* is calculated by halved-distance iterations until the estimate (the left side of Equation 4 and 5) differed from the target with less than 0.1‰. The population is harvested by stepping the SOM one year with the catch being determined by Baranov’s catch equation:
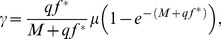
(6)in which we use the actual *q* of the SOM.

### Sensitivity to error in catchability

We keep the estimated catchability 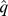 in Equation (2) and (3) constant and unbiased in our base runs, but fishery dependent catchability may in reality be biased and vary over time. Such uncorrelated errors and biases in catchability will contribute to error or bias in the assessment. Our base model already has assessment error (CV = 21%, as described in *Assessment and priors*) for supervised HCRs, but unsupervised BRs do not (Table 1). Unsupervised BRs do not use separate assessments, but on the other hand they will be affected by error or bias in 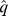. We chose to compare supervised *F*-targeted CR and unsupervised *F*-targeted BR because the former is only affected by the error in the assessed stock size, *µ*, whereas the latter is affected only by the error in 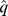. We also explored the sensitivity to correlated biases in 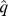
and *µ* by changing their values ±20%. Simulations ran for 41,000 generations and data from the last 40,000 were used for the analyses.

### Statistical analyses

Unsupervised CR expectedly led to population crashes within a few decades or less. We therefore excluded them in the statistical analyses, which were performed using STATISTICA software (Statsoft, Tulsa, Oklahoma). General linear models (GLM) were used for three-way ANOVAs, testing the effects of supervision mode, regulation type and target type on yield, fishing mortality, spawning stock biomass, and post-harvest population size. Fourier analyses were performed on 1,000 year data series, tapered by 15% and padded to the length power of 2. The period length in years with the highest spectral density was identified after applying Hamming weights with a data window of size 7. Test of significant deviation from white noise was performed using Kolmogorov-Smirnov deviation (*d*) statistics, Table Y, *ρ* = 0.5, n>100 in Rohlf & Sokal [41].

## Results

### Yield

**Figure 2:**
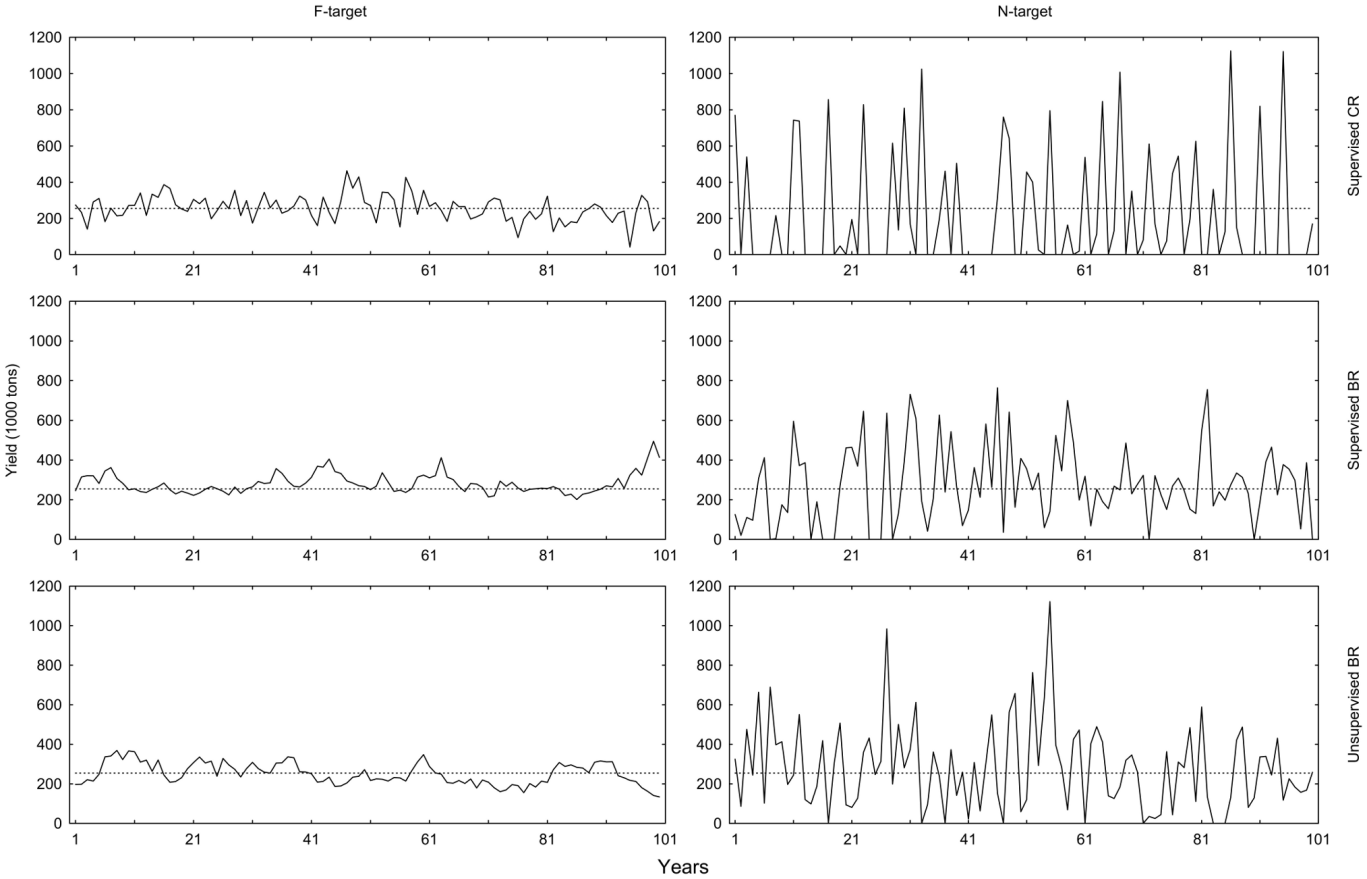
Yield over 100 years for different HCRs. The dashed reference line is the MSY = 254.4 thousand tons obtained from the *F*_MSY_-analysis during controlled *F*.

**Figure 3:**
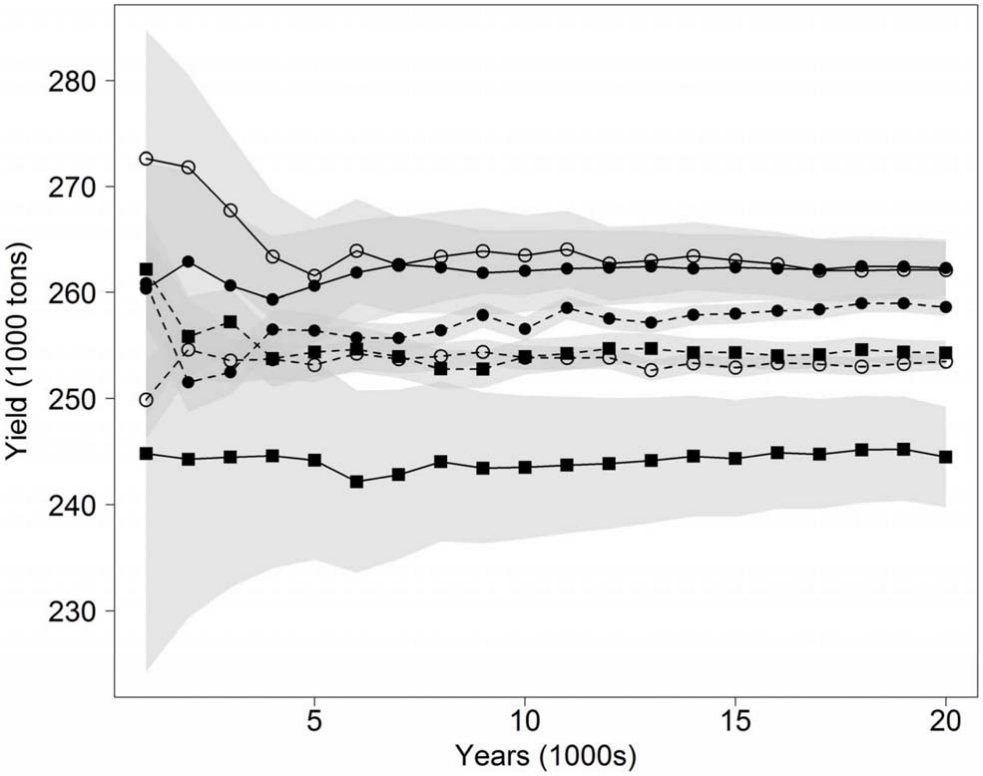
Average yields and confidence intervals as functions of increasing length of simulation in years. Filled symbols denote supervised and open symbols unsupervised harvest control. Squares denote CR and circles BR. Solid lines denote *N*-target management whereas hatched lines denote *F*-targeted management.

Mean yield in 100-year simulations is significantly higher with BR than with CR although the differences are small (Fig. 2, Table 2, 3). With an increasing length of the time series the confidence intervals narrow (Fig. 3). Analysis of 20,000 years reveals more clearly that BRs operated with *N*-target give the highest average yields, even though the largest differences in means are less than 10% (Table 2, 4). Supervised and unsupervised BRs give higher yields (262 thousand tons) than the reference MSY from the *F*_MSY_ -analysis (254 thousand tons; Table 2, 3, 4, 5). Contrary to the small differences in mean yields, the CV in yields is clearly affected by the choice of HCR. In general, using *F*-targets gave less temporal variation than using *N*-targets (Fig. 2, Table 2, 3, 4, 5). There is also an effect of regulation type with BR, giving less variation than CR (Fig. 2, Table 3, 4). However, supervised BR and unsupervised BR have the same CV regardless of target (Table 5).

**Table 2:** Mean, standard deviation and CV (%) of annual yield.

	100 yrs			20,000 yrs		
	mean	SD	CV	mean	SD	CV
CR_F_^s^	256.6	72.0	28.1	254.3	83.9	33.0
CR_N_^s^	220.3	322.4	146.3	244.5	343.0	140.3
BR_F_^s^	282.8	50.6	17.9	258.6	58.9	22.8
BR_N_^s^	272.5	198.8	73.0	262.3	195.2	74.4
BR_F_^u^	251.8	55.0	21.9	253.5	57.6	22.7
BR_N_^u^	281.9	218.9	77.7	262.1	196.5	75.0

Two time series, one of 100 years and one of 20,000 years are presented. Mean values are given in thousands of tons. MSY from the *F*_MSY_ -analysis is 254.4 thousand tons.

**Table 3:** ANOVA-table showing effects of supervision, regulation type, target type, and target type interacting with supervision/regulation type on annual yield.

100 yrs	SS	DF	MS	F	p
Supervision	11,691	1	11,691	0.35	0.556
Regulation type	153,773	1	153,773	4.56	0.033
Target type	2,831	1	2,831	0.08	0.772
Superv.×Target	40,995	1	40,995	1.22	0.271
Regul.×Target	16,783	1	16,783	0.50	0.481
Error	20,018,836	594	33,702		

Data is from model simulations over 100 yrs.

**Table 4:** ANOVA-table showing effects of supervision, regulation type, target type, and target type interacting with supervision/regulation type on annual yield.

	SS	DF	MS	F	p
Supervision	141,961	1	141,961	4.16	0.041
Regulation type	1,582,157	1	1,582,157	46.40	<0.001
Target type	58,951	1	58,951	1.73	0.189
Superv×Target	120,612	1	120,612	3.54	0.060
Regul×Target	1,582,157	1	1,582,157	46.40	<0.001
Error	4,091,815,000	119,994	34,100		

Data is from model simulations over 20,000 yrs.

**Table 5:** Pair-wise test of CV in yield for the 100 year series, applying the F-statistics of testing differences in the variance of annual yield.

	CR_F_^s^	CR_N_^s^	BR_F_^s^	BR_N_^s^	BR_F_^u^	BR_N_^u^
CR_F_^s^		<0.001	<0.001	<0.001	0.007	<0.001
CR_N_^s^	27.205		<0.001	<0.001	<0.001	<0.001
BR_F_^s^	2.455	66.785		<0.001	0.024	<0.001
BR_N_^s^	6.764	4.022	16.605		<0.001	0.268
BR_F_^u^	1.647	44.809	1.490	11.141		<0.001
BR_N_^u^	7.661	3.551	18.807	1.133	12.619	

F-values are presented in the lower left triangle and p-values in the upper right.

### Fishing mortality

**Figure 4:**
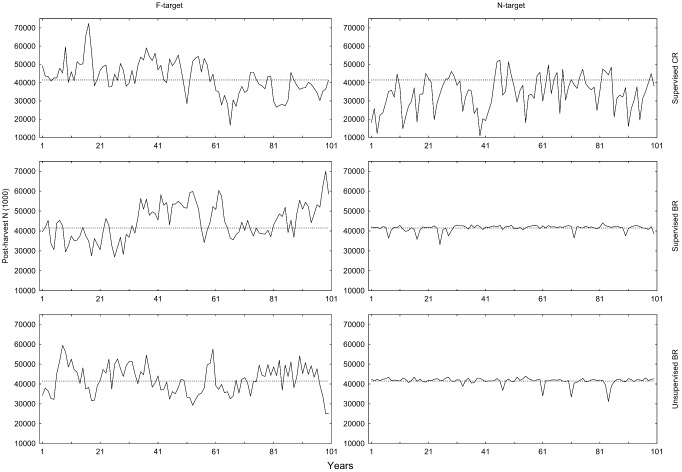
Fishing mortality (*F*) for 100 years for different HCRs. The dashed reference line is the *F*_MSY_ = 0.17 obtained from the *F*_MSY_-analysis during controlled *F*.

There is no significant difference in mean fishing mortality (*F*) due to any effect over 100 years (Table 6, Table 7). All HCRs reach the *F*-target of 0.17 as their mean, except supervised CR with *N*-target, which has a mean *F* of 0.20. Although the mean *F*s are very similar across HCRs, the differences in CV are more pronounced. Supervised CR with *N*-target exhibits the highest CV of 158% (Table 6). *F*-targeted supervised CR has a CV of 25% (Table 6, Fig. 4). Since catchability is constant here, *F* is proportional to fishing effort. It is therefore not surprising that *F*-targeted HCRs result in less variation than *N*-targeted HCRs. *F*-targeted BRs, supervised and unsupervised, show very high precision in reaching the target (Fig. 4), the CV being as low as 0.1% (Table 6). As expected, *N*-targeted BRs exhibit higher CVs (68% unsupervised and 70% supervised; Table 6). In addition, it is only the CV between supervised and unsupervised BRs that does not differ significantly from each other (Table 8).

**Table 6:** Mean, standard deviation and CV (%) of annual fishing mortality (*F*) of 100 years of simulated fishing.

	mean	SD	CV
CR_F_^s^	0.17	0.043	25.2
CR_N_^s^	0.20	0.325	158.4
BR_F_^s^	0.17	0.000	0.1
BR_N_^s^	0.17	0.118	70.1
BR_F_^u^	0.17	0.000	0.1
BR_N_^u^	0.17	0.119	68.4

*F*_MSY_ from the *F*_MSY_ -analysis is 0.17.

**Table 7:** ANOVA-table showing effects of supervision, regulation type, target type, and target type interacting with supervision/regulation type on annual fishing mortality.

	SS	DF	MS	F	p
Supervision	0.00047	1	0.00047	0.02	0.886
Regulation type	0.03166	1	0.03166	1.40	0.236
Target type	0.02945	1	0.02945	1.31	0.254
Superv×Target	0.00046	1	0.00046	0.02	0.886
Regul×Target	0.03265	1	0.03265	1.45	0.229
Error	13.39279	594	0.02255		

Data is from model simulations over 100 yrs.

**Table 8:** Pair-wise test of CV-values applying the F-statistics of testing differences in the variance of annual fishing mortality.

	CR_F_^s^	CR_N_^s^	BR_F_^s^	BR_N_^s^	BR_F_^u^	BR_N_^u^
CR_F_^s^		<0.001	<0.001	<0.001	<0.001	<0.001
CR_N_^s^	39.520		<0.001	<0.001	<0.001	<0.001
BR_F_^s^	67,742	2,677,131		<0.001	0.066	<0.001
BR_N_^s^	7.731	5.112	523,678		<0.001	0.404
BR_F_^u^	49,987	1,975,470	1.355	386,425		<0.001
BR_N_^u^	7.361	5.369	498,613	1.050	367,930	

F-values are presented in the lower left triangle and p-values in the upper right.

### Post-harvest population size and SSB

**Figure 5:**
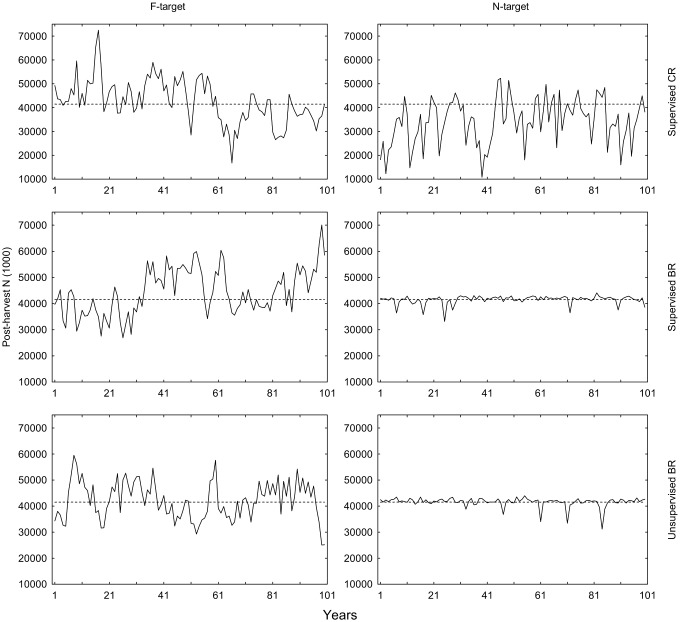
Post-harvest *N*-values for different HCRs in simulations over 100 years. The dashed reference line is the *N*-target = 41,491 million obtained from the *F*_MSY_ analysis during controlled *F*.

Looking at post-harvest population size (*N*’), the harvest controls operating on *N*-targets are aiming for 41.5 billion individuals at the end of the year. The two *N*-targeted BRs reach this target with little variation between years (4% CV; Fig. 5, Table 9, 10, 11), whereas *N*-targeted CR leads to over-fishing in the sense that *N*’ is on average 17% below target. In contrast to the *N*-targeted regulation types, there is a small difference in *N*’ between *F*-targeted BR and CR. This explains the significant interaction between regulation type and target type in the ANOVA (Table 10). Supervision has no significant effect on the mean *N*’ (Table 10), nor the CV (Table 11).

**Table 9:** Mean (Million), standard deviation and CV (%) of annual post-harvest population size, *N*’, of 100 years of simulated fishing.

	mean	SD	CV
CR_F_^s^	42,620	9,201	21.6
CR_N_^s^	34,282	9,470	27.6
BR_F_^s^	44,391	8,798	19.8
BR_N_^s^	41,508	1,616	3.9
BR_F_^u^	42,270	7,248	17.1
BR_N_^u^	41,569	1,840	4.4

*N*-target from the *F*_MSY_ -analysis is 41,491 million.

**Table 10:** ANOVA-table showing effects of supervision, regulation type, target type, and target type interacting with supervision/regulation type on annual post-harvest population size.

	SS	DF	MS	F	p
Supervision	1.06E+08	1	1.06E+08	2.1	0.153
Regulation type	2.02E+09	1	2.02E+09	39.1	<0.001
Target type	2.44E+09	1	2.44E+09	47.2	<0.001
Superv×Target	1.19E+08	1	1.19E+08	2.3	0.130
Regul×Target	7.44E+08	1	7.44E+08	14.4	<0.001
Error	3.07E+10	594	5.17E+07		

Data is from model simulations over 100 yrs.

**Table 11:** Pair-wise test of CV-values applying the F-statistics of testing differences in the variance of annual post-harvest population size.

	CR_F_^s^	CR_N_^s^	BR_F_^s^	BR_N_^s^	BR_F_^u^	BR_N_^u^
CR_F_^s^		0.007	0.198	<0.001	0.011	<0.001
CR_N_^s^	1.637		0.001	<0.001	<0.001	<0.001
BR_F_^s^	1.187	1.943		<0.001	0.076	<0.001
BR_N_^s^	30.736	50.318	25.903		<0.001	0.103
BR_F_^u^	1.585	2.595	1.336	19.387		<0.001
BR_N_^u^	23.800	38.964	20.058	1.291	15.012	

F-values are presented in the lower left triangle and p-values in the upper right.

**Figure 6:**
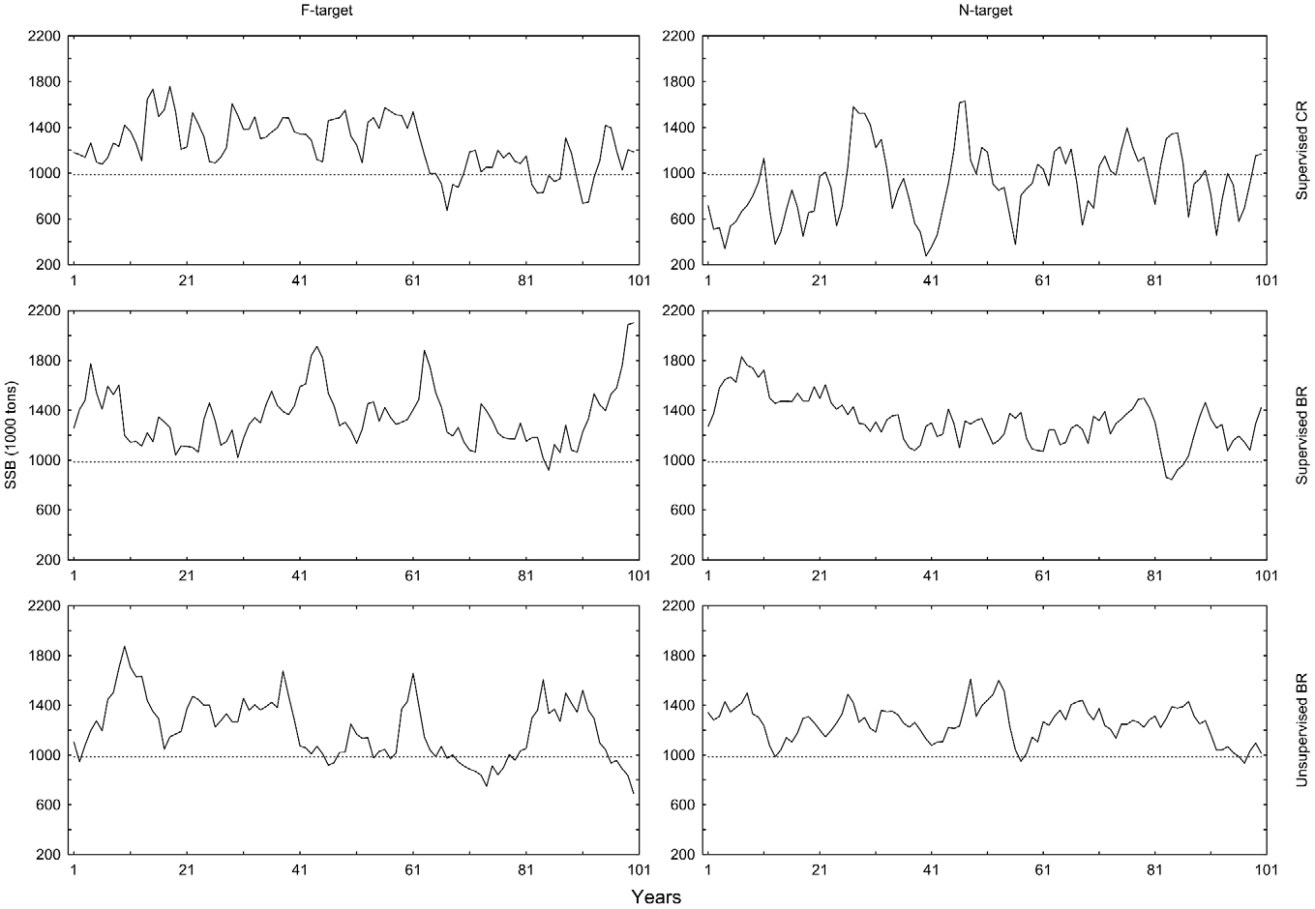
Spawning stock biomass (SSB) for different HCRs in simulations over 100 years. The dashed reference line is the *B_trigger_* = 988 thousand tons. It is the 2.5% lower percentile of SSB obtained from the *F*_MSY_-analysis during controlled *F*.

The mean SSBs differ significantly by regulation type, harvest type and supervision (Table 12). BR SSBs are on average larger than CR SSBs (Fig. 6, Table 13, 13). Supervised CR with *N*-target has the lowest mean and is below B_trigger_ in 50% of the years (Table 13, Fig. 6). Supervised BRs has on average larger SSBs than unsupervised, and the BRs operating with *N*-targets surpass the B_trigger_ less frequently than when operating with *F*-targets (Table 13, Fig. 6). The lower CV for BRs operating with *N*-target (Table 13, 14) seems to be the main reason for this result.

**Table 12:** ANOVA-table showing effects of supervision, regulation type, target type, and target type interacting with supervision/regulation type on annual SSB.

	SS	DF	MS	F	p
Supervision	1 054 971	1	1 054 971	19.8	<0.001
Regulation type	6 757 878	1	6 757 878	126.9	<0.001
Target type	2 008 542	1	2 008 542	37.7	<0.001
Superv×Target	164 153	1	164 153	3.1	0.080
Regul×Target	2 271 205	1	2 271 205	42.6	<0.001
Error	31 642 602	594	53 270		

Data is from model simulations over 100 yrs.

**Table 13:** Mean in thousand tons, standard deviation and CV (%) of annual SSB of 100 years of simulated fishing.

	mean	SD	CV	<B_trigger_ (%)
CR_F_^s^	1 240	230.0	18.6	26.1
CR_N_^s^	906	308.6	34.1	49.9
BR_F_^s^	1 349	232.8	17.3	18.3
BR_N_^s^	1 317	192.4	14.6	5.6
BR_F_^u^	1 206	245.1	20.3	20.6
BR_N_^u^	1 255	142.3	11.3	5.9

The percentage of years the SSB surpassed the *B_trigger_* (*B_trigger_ = *998 thousand tons), is given in the rightmost column.

**Table 14:** Pair-wise test of CV in yield for the 100 year series, applying the F-statistics of testing differences in the variance of annual SSB.

	CR_F_^s^	CR_N_^s^	BR_F_^s^	BR_N_^s^	BR_F_^u^	BR_N_^u^
CR_F_^s^		<0.001	0.236	0.009	0.183	<0.001
CR_N_^s^	3.368		<0.001	<0.001	<0.001	<0.001
BR_F_^s^	1.156	3.894		0.049	0.053	<0.001
BR_N_^s^	1.614	5.434	1.396		0.001	0.006
BR_F_^u^	1.200	2.807	1.387	1.936		<0.001
BR_N_^u^	2.678	9.018	2.316	1.660	3.213	

F-values are presented in the lower left triangle and p-values in the upper right.

### Bias and error in harvest rates

**Figure 7:**
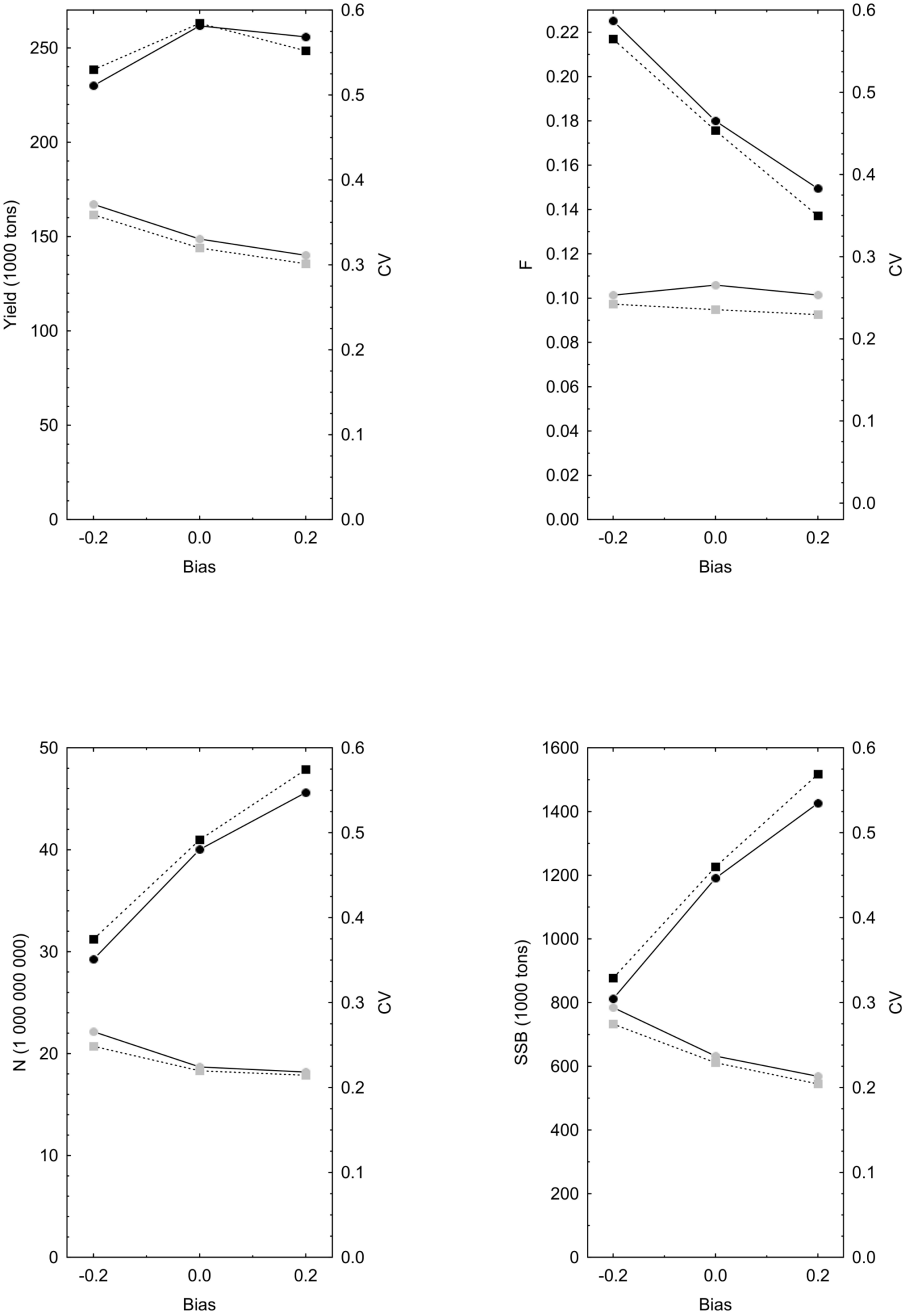
A comparison of yield, fishing mortality (*F*), post-harvest stock size (*N*’) and spawning stock biomass (SSB) when random errors of the same CV are applied to both the stock size assessment (*µ*) and the estimated catchability parameter (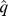). Relative positive and negative biases applied to 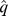 and *µ* are denoted on the x-axis. Circles denote results from *F*-targeted unsupervised BR and squares *F*-targeted supervised CR. Black symbols denote the value (left axis) and grey symbols the CV of the effect-variable (right axis).

So far we can summarize the difference between CR and BR as being BR’s ability to reduce the variance in the target variable (*F* or *N*’; Tables 3, 4). Yield is partially dependent on *F*, and SSB on *N*’, and thus the variance of these variables is also reduced, although to a lesser degree (Tables 2, 5). These results are based on the assumption of a constant and un-biased estimate of catchability (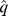). When random error is added to the catchability (*q*) of the same magnitude as the error in *µ*, the CV in γ and *F* for the unsupervised *F*-targeted BR become similar to supervised *F*-targeted CR (Fig. 7). The two *F*-targeted harvest rules also perform very similar in response to the off-set bias in *µ* and 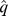. Hence, supervised CR and unsupervised BR behave similarly when error and bias in *µ* and *q* are of the same relative magnitude.

### Temporal variation in population size

**Figure 8:**
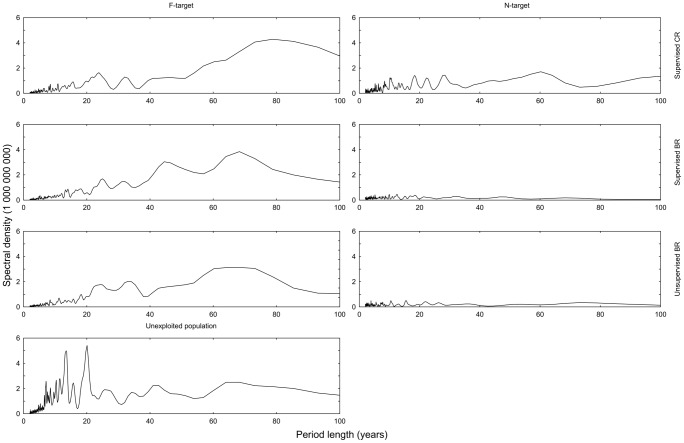
Spectral densities of Fourier analysis of 1,000 years of pre-harvest population size under various HCRs. Period lengths from 0 to 100 years are shown (depicted on the abscissa).

We found that all HCRs decrease the amplitude of temporal variation in pre- and post-harvest population numbers, except for post-harvest numbers under supervised *N*-targeted CR, at which the variation is indifferent from the unexploited population (Table 15). The BR is effective in reaching the *N*-target, and hence reduces the CV markedly in the post-harvest population size (Fig. 8, Table 15). The effect is still evident in the pre-harvest population size, in which the inclusion of recruits from the foregoing year increases the variation (Table 15).

**Table 15:** Coefficient of variation (CV) of pre- and post-harvest population numbers compared with the unexploited population.

	Pre-harvest *N*			Post-harvest *N*		
	CV	F-value	p	CV	F-value	p
CR_F_^s^	21.6	1.52	<0.001	22.0	1.42	<0.001
CR_N_^s^	24.5	1.18	<0.001	26.1	1.00	n.s.
BR_F_^s^	21.2	1.57	<0.001	21.2	1.53	<0.001
BR_N_^s^	14.8	3.25	<0.001	4.6	33.01	<0.001
BR_F_^u^	20.8	1.64	<0.001	20.7	1.60	<0.001
BR_N_^u^	15.1	3.12	<0.001	4.7	30.58	<0.001
Unexpl. Pop.	26.6			26.1		

CV is from simulations of 10,000 years. F-value is the ratio of squared CVs (i.e. comparison of standardized variances).

The unexploited population exhibited a very pronounced periodicity of about 20 years (Fig. 8, Table 16). There is only uncorrelated variation in our operating model, which means that any periodicity is due to the demographic parameters and structure of the population. The BRs with *N*-target remove much of this periodicity and puts it closer to white noise (Lower K-S d-values, Table 16). HCRs with *F*-target increase the dominating period length to about 70–80 years (Table 16). Supervised CR with *F*-target, a simplified version of the HCR currently used, reduces the amplitude of variation in both pre- and post-harvested population size (Table 15). At the same time, it increases the dominating period length from 20 years in the unexploited population to about 80 years in the exploited (pre-harvest population size, Table 16).

**Table 16:** Fourier analysis of pre-harvest population numbers in series of 1,000 years of fishing with alternative HCRs.

HCR	Period max	Density	K-S d	p
CR_F_^s^	78.769	4.29E+09	0.5013	<0.01
CR_N_^s^	60.235	1.71E+09	0.3152	<0.01
BR_F_^s^	68.267	3.84E+09	0.5370	<0.01
BR_N_^s^	12.488	4.62E+08	0.0929	<0.01
BR_F_^u^	68.267	3.13E+09	0.5368	<0.01
BR_N_^u^	15.515	5.10E+08	0.0716	<0.01
Unexpl. Pop.	20.078	5.42E+09	0.4995	<0.01

Period length with maximum spectral density is given in years along with density value. Kolmogorov-Smirnov d-value for deviation from white noise is given with its p-value.

## Discussion

We have suggested a new approach to fisheries regulation, which we name *Bayesian regulation* (BR), and made a basic evaluation of its properties in relation to catch-only regulation. The idea of BR is adopted from Bayesian foraging theory, in which patch-foragers are assumed to rely on a giving-up rule based on prior information and sampling information [29], [30], [42]. The Bayesian foraging theory has been studied extensively, including the derivation of the probability function used and that cumulative catch and effort are sufficient information variables [29]. The BR uses the uncertainty of the stock size estimate as a parameter, which is rarely the case in current management [43]–[45], and which has been requested for some time [46]. The general applicability of the BR is emphasized by the fact that catch-only regulation, (CR), is a special case of BR ― i.e. when there is no uncertainty in the prior estimate of the stock size from an assessment or survey. We have analyzed three aspects of harvest controls: (i) the relevance of supervision (with or without stock size assessment), (ii) operational target type (constant fishing mortality, *F*, or constant stock size, *N*), (iii) type of regulation (catch only* = *CR, or Bayesian evaluation of combined catch and effort* = *BR), and their effect on mean and variance in yield and stock size.

### The effect of fishing on stock dynamics

There is an ongoing discussion about whether fishing leads to increased temporal variability in stock size compared to unexploited populations [4], [14]. Observations from California, USA, corroborate the view that fishing magnifies fluctuations in fish abundance, driven by increased intrinsic growth rates as a response to higher mortality [4]. Models without age-structure show an increased variability in population abundance as a consequence of exploitation [14]. In a structured model, fishing can either increase or decrease the stock size variability [15]. We find that the CV of population size has been reduced in all investigated HCRs, and especially with *N*-targeted BR. The different effects of fishing between our and the previous studies may depend on our implementation of errors in assessment, whereas earlier studies implemented perfect fishing mortality.

### The comparison of Bayesian- and catch-regulation

We expected *N*-targeted BR to give the highest mean yields, because of its ability to maintain the population in the most productive state. It turned out to return the highest mean yield, but the differences between HCRs are small. The CV of fishing mortality and the population size is, however, much more affected using BR compared to CR. The advantage of *F*-targeted BR is seen in a reduced variation in fishing mortality and yield. Similarly, the *N*-targeted BR is associated with less variation in post-harvest population size and SSB. Under some conditions CR can be a blunt harvest control instrument; populations inevitably go extinct if not supervised, and it exhibits the largest CV in yield, *F* and SSB of all harvest controls when operated with *N*-target. For the better, *F*-target supervised CR is a commonly applied harvest control, e.g. within the European Union, and exhibits a smaller CV than *N*-targeted CR in all fisheries and stock variables investigated. Choosing BR, a manager has to consider the trade-off between the pros and cons of an *F*- versus an *N*-targeted fishery, since targeting one variable pushes the variability to the other [47]. From a fisheries perspective, *F*-targeted BR can appear as the most preferable HCR: low variability in effort (costs) and yield (income) enables a more stable economy, and an even process load is more manageable for the food industry [47]. At the same time, single-stock *F*-targeted BR can potentially yield a conflict of interest between fishers, and may be less desirable when taking into account the ecological interaction between different species of fish. *N*-targeted BR, on the other hand, gives low variation in population size, which means lower risks of extinction and smaller indirect effects of variability on prey and predator species, which may also be subject to commercial fishing. The stable post-harvest population size of *N*-targeted BR, in combination with the inherent variation in the productivity of the population causes a highly variable yield.

Our results show that *F*-targeted BRs come close to resolving the trade-off between a high long-term yield and the stable fishing effort and yield acknowledged by some authors [20], [35]. Although giving a slightly lower average yield than BRs with *N*-target, the yield is higher than with CR and the fluctuations in the yield are reduced considerably.

The ability of the BR to control the target variable depends on the reliability of the estimated fishery-dependent catchability. We have shown, as a rule of thumb, that if the CV of the error in catchability is as large as the error CV in the stock size assessment, the unsupervised BR performs similar to the supervised CR. Lack of knowledge makes it difficult to say in general which of the errors is the largest, in fishery-dependent catchability or in assessments. In the case of the Newfoundland cod, CPUE increased when stock abundance declined due to hyper-aggregation of the fish [40]. Such deceitful behavior of CPUE warrants close attention, and neither BRs nor CRs are immune to it. It is possible to estimate catchability accurately when we know the fishing gear, the fishers’ behavior, and the fish dispersal pattern. One could say that a transfer from CR to BR would require a shift of focus by managers from surveys and assessments to gear efficiency, behavior of fish and fishing fleets.

### HCRs’ effects on period lengths of stock size variation

Not only the amplitude of population variation, but also the frequency of the variation has consequences for the population [48]. The induced periodic population variability in predator-prey interactions is well known in ecology [49], but the result of the dynamic interaction between the fish stock and the harvest control is much less studied [15]. It has been hypothesized that populations truncated by fishing have a shorter inherent periodicity, and track environmental fluctuations more closely. Given the assumption that unharvested populations fluctuate at longer frequencies than the environment, this could be the reason for increased amplitude of population variation [4]. Since populations have inherent dynamics controlled by age-specific demographic parameters, one can conclude that populations truncated by harvest should exhibit fluctuations of shorter period length than unexploited ones. In our study, this is true for the *N*-targeted BRs, but for the other harvest controls the period length of the fluctuations increase. For *F*-targeted fisheries, stock size deviations from equilibrium delays return times because of a positive feed-back: enlarged populations face reduced mortality and reduced populations are subject to increased mortality. If stocks exhibit long-wave fluctuations with period lengths of 60–80 years due to fishing as indicated with *F*-targeted CR fisheries, the stress it imposes on ecosystems warrants more attention in future studies and consideration in the choice of method for harvest control.

### Potential application of Bayesian regulation

The Bayesian regulation of fisheries modeled here is highly idealized. Real, well-informed management processes are far more complex, and may have sources of error not accounted for. We want to emphasize the potential of BR and stimulate ideas in how it might be implemented. The concept of BR can be developed further, for example to include a Bayesian estimate of the catchability parameter in the probability function (Equation 1). BR has the advantage that, within a year, it can give much higher precision in reaching the management target compared to CR. For instance, stock control with quantitative assessment and forecast can utilize a Bayesian HCR for the harvest year. New assessment methods that calculate a confidence interval for stock size estimate, such as SAM (Nielsen and Berg unpublished), is ideal as they provide priors for a Bayesian regulation.

Another useful property of BR is the ability to work without other assessments, since it is an assessment in itself. It can be an option for the more than 99% of fished species which lack assessments because of the costs and other requirements for collecting the necessary data [50]. An application of BR would still require daily reporting of catches, effort and gear used. But with such a system in place, Bayesian estimates of stock size and fishing mortality could be provided daily, with fishers and managers more up-to-date with current stock status.

## Supporting Information

Appendix S1 Derivation of the generalized Bayesian estimate of remaining prey population.
(PDF)

Appendix S2 Description of the stochastic operating model of the Baltic Sea herring.
(PDF)

Code S1 R-code file “control.r”. The file contains the highest levels of instructions like regulation type, target type etc. In addition, the targets and other constants are set here. Running the file in R with the three other files in the same folder will produce and plot the results of the specified setup.
(R) Click here for additional data file.

Code S2 R-code file “MSE.r”. The file contains most of the actual code of the management strategy evaluation procedure. Parameters like simulation length (number of generations), predator spawning stock biomass, and other parameters are set in this file.
(R) Click here for additional data file.

Code S3 R-code file “BR.r”. The file contains the algorithm to produce the fishing mortality that the BR suggests. The error and bias of A are set here, as is the tolerance of the algorithm. This code is used both for CR and BR where catch only regulation results are produced by having the variance set to zero.
(R) Click here for additional data file.

Code S4 R-code file “OPMOD.r”. The file contains the stochastic operating model. The parameters that are more directly linked to the population are set here, as are also the initial values of the population numbers-at-age and weights-at-age. The functions that describe mortality, reproduction, growth, and the weight of one year olds are defined here.
(R) Click here for additional data file.

## References

[1] Virtala M, Kuikka S, Arjas E (1998) Stochastic virtual population analysis. ICES J Mar Sci 55: 892–904.

[2] Mäntyniemi S, Kuikka S, Rahikainen M, Kell LT, Kaitala V (2009) The value of information in fisheries management: North Sea herring as an example. ICES J Mar Sci 66: 2278–2283.

[3] Polasky S, Carpenter SR, Folke C, Keeler B (2011) Decision-making under great uncertainty: environmental management in an era of global change. Trends Ecol Evol 26: 398–404. 21616553 10.1016/j.tree.2011.04.007

[4] Anderson CNK, Hsieh C, Sandin SA, Hewitt R, Hollowed A, et al (2008) Why fishing magnifies fluctuations in fish abundance. Nature 452: 835–839. 18421346 10.1038/nature06851

[5] Jonzén N, Ripa J, Lundberg P (2002) A Theory of Stochastic Harvesting in Stochastic Environments. Am Nat 159: 427–437. 18707427 10.1086/339456

[6] Getz WM (1985) Optimal and Feedback Strategies for Managing Multicohort Populations. J Optimiz Theory App 46: 505–514.

[7] Walters CJ, Ludwig D (1981) Effects of Measurement Errors on the Assessment of Stock-Recruitment Relationships. Can J Fish Aquat Sci 38: 704–710.

[8] Hjerne O, Hansson S (2001) Constant catch or constant harvest rate? Baltic Sea cod (*Gadus morhua* L.) fishery as a modelling example. Fish Res 53: 57–70.

[9] Aps R, Fetissov M, Holmgren N, Norrström N, Kuikka S (2011) Central Baltic Sea herring: effect of environmental trends and fishery management. In: Villacampa Y, Brebbia CA, editors. Ecosystems and Sustainable Development VIII. Boston: WIT Press Southampton. pp. 69–80.

[10] Hightower JE, Grossman GD (1985) Comparison of constant effort harvest policies for fish stocks with variable recruitment. Can J Fish Aquat Sci 42: 982–988.

[11] Koonce JF, Shuter BJ (1987) Influence of Various Sources of Error and Community Interactions on Quota Management of Fish Stocks. Can J Fish Aquat Sci 44: s61–s67.

[12] Murawski SA, Idoine JS (1989) Yield Sustainability under Constant-Catch Policy and Stochastic Recruitment. T Am Fish Soc 118: 349–367.

[13] Hsieh C, Reiss CS, Hunter JR, Beddington JR, May RM, et al (2006) Fishing elevates variability in the abundance of exploited species. Nature 443: 859–862. 17051218 10.1038/nature05232

[14] Shelton AO, Mangel M (2011) Fluctuations of fish populations and the magnifying effects of fishing. PNAS 108: 7075–7080. 21482785 10.1073/pnas.1100334108 PMC3084066

[15] Wikström A, Ripa J, Jonzén N (2012) The role of harvesting in age-structured populations: disentangling dynamic and age-truncation effects. Theor Popul Biol 82: 348–354. 22227065 10.1016/j.tpb.2011.12.008

[16] Kell LT, Pilling GM, Kirkwood GP, Pastoors M, Mesnil B, et al (2005) An evaluation of the implicit management procedure used for some ICES roundfish stocks. ICES J Mar Sci 62: 750–759.

[17] Kraak SBM, Kelly CJ, Codling EA, Rogan E (2010) On scientists’ discomfort in fisheries advisory science: the example of simulation-based fisheries management-strategy evaluations. Fish and Fisheries 11: 119–132.

[18] Rochet M-J, Rice JC (2009) Simulation-based management strategy evaluation: ignorance disguised as mathematics? ICES J Mar Sci 66: 754–762.

[19] Stephenson R, Peltonen H, Kuikka S, Pönni J, Rahikainen M, et al. (2001) Linking Biological and Industrial Aspects of the Finnish Commercial Herring Fishery in the Northern Baltic Sea. In: Funk F, Blackburn J, Hay D, Paul AJ, Stephenson R et al.., editors. Herring: Expectations for a New Millennium. Fairbanks: University of Alaska Sea Grant. pp.741–760.

[20] May RM, Beddington JR, Horwood JW, Shepherd JG (1978) Exploiting natural populations in an uncertain world. Mathematical Biosciences 42: 219–252.

[21] Hilborn R, Mangel M (1997) The ecological detective. Princeton: Princeton University Press.

[22] Dall SRX, Giraldeau L-A, Olsson O, McNamara JM, Stephens DW (2005) Information and its use by animals in evolutionary ecology. Trends Ecol Evol 20: 187–193. 16701367 10.1016/j.tree.2005.01.010

[23] McNamara JM, Green RF, Olsson O (2006) Bayes’ theorem and its applications in animal behaviour. Oikos 112: 243–251.

[24] Valone TJ (1991) Bayesian and prescient assessment: foraging with pre-harvest information. Anim Behav 41: 569–577.

[25] Charnov EL (1976) Optimal foraging, the marginal value theorem. Theor Popul Biol 9: 129–136. 1273796 10.1016/0040-5809(76)90040-x

[26] Olsson O, Holmgren NMA (1999) Gaining ecological information about Bayesian foragers through their behaviour. I. Models with predictions. Oikos 87: 251–263.

[27] Olsson O, Wiktander U, Holmgren NMA, Nilsson SG (1999) Gaining ecological information about Bayesian foragers through their behaviour. II. A field test with woodpeckers. Oikos 87: 264–276.

[28] Green RF (1980) Bayesian birds: a simple example of Oaten’s stochastic model of optimal foraging. Theor Popul Biol 18: 244–256.

[29] Iwasa Y, Higashi M, Yamamura N (1981) Prey distribution as a factor determining the choice of optimal foraging strategy. Am Nat 117: 710–723.

[30] Olsson O, Holmgren NMA (1998) The survival-rate-maximizing policy of Bayesian foragers: wait for good news! Behav Ecol. 9: 345–353.

[31] Holmgren NMA, Norrström N, Aps R, Kuikka S (2012) MSY-orientated management of Baltic Sea herring (Clupea harengus) during different ecosystem regimes. ICES J Mar Sci 69: 257–266.

[32] ICES (2011) Report of the Workshop on Implementing the ICES Fmsy Framework (WKFRAME-2), 10–14 February 2011, ICES, Denmark. ICES CM 2011/ACOM 33: 110 pp.

[33] European Commission (2002) On the conservation and sustainable exploitation of fisheries resources under the Common Fisheries Policy. Council regulation (EC) No 2371/2002.

[34] Marchal P, Horwood J (1998) Increasing fisheries management options with a flexible cost function. ICES J Mar Sci 55: 213–227.

[35] Pelletier D, Laurec A (1992) Management under uncertainty: defining strategies for reducing overexploitation. ICES J Mar Sci 49: 389–401.

[36] ICES (2009) Report of the ICES Advisory Committee, 2009. ICES Advice, 2009. Book 8, 132 pp.

[37] ICES (2012) Report of the Workshop 3 on Implementing the ICES Fmsy Framework, 9–13 January 2012, ICES, Headquarters. ICES CM 2012/ACOM 39: 1–33.

[38] ICES (2012) Report of the ICES Advisory Committee 2012. ICES Advice, 2012. Book 8, 158 pp.

[39] Valone TJ, Brown JS (1989) Measuring Patch Assessment Abilities of Desert Granivores. Ecology 70: 1800–1810.

[40] Rose GA, Kulka DW (1999) Hyperaggregation of fish and fisheries: how catch-per-unit-effort increased as the northern cod (Gadus morhua) declined. Can J Fish Aquat Sci 56: 118–127.

[41] Rohlf FJ, Sokal RR (1995) Statistical tables. New York: W.H. Freeman & Co.

[42] Green RF (1984) Stopping rules for optimal foragers. Am Nat 123: 30–43.

[43] Patterson KR (1999) Evaluating uncertainty in harvest control law catches using Bayesian Markov chain Monte Carlo virtual population analysis with adaptive rejection sampling and including structural uncertainty. Can J Fish Aquat Sci 56: 208–221.

[44] Prager MH, Porch CE, Shertzer KW, Caddy JF (2003) Targets and limits for management of fisheries: a simple probability-based approach. N Am J Fish Manage 23: 349–361.

[45] Shertzer KW, Prager MH, Williams EH (2008) A probability-based approach to setting annual catch levels. Fish Bull 106: 225–232.

[46] Patterson K, Cook R, Darby C, Gavaris S, Kell L, et al (2001) Estimating uncertainty in fish stock assessment and forecasting. Fish and Fisheries 2: 125–157.

[47] Getz WM, Francis RC, Swartzman GL (1987) On Managing Variable Marine Fisheries. Can J Fish Aquat Sci 44: 1370–1375.

[48] Ripa J, Lundberg P (1996) Noise colour and the risk of population extinctions. Proc R Soc London B 263: 1751–1753.

[49] Yodzis P (1991) Introduction to theoretical ecology. New York: Harper & Row.

[50] Costello C, Ovando D, Hilborn R, Gaines SD, Deschenes O, et al (2012) Status and Solutions for the World’s Unassessed Fisheries. Science 338: 517–520. 23019613 10.1126/science.1223389

