# Supplementary material for: A Concept of Bayesian Regulation in Fisheries Management
Source: PLoS One. 2014 Nov 3;9(11):e111614. doi: 10.1371/journal.pone.0111614 (PMC4218784; doi:10.1371/journal.pone.0111614)
Supplement: Appendix S1 — Derivation of the generalized Bayesian estimate of remaining prey population. (PDF) [file pone.0111614.s001.pdf]

Supporting Information - Appendix S1

Iwasa et al. [1] showed that the remaining number of food items in a patch (r) after t time units of random search with efficiency A, during which n items have been found can be described as:

$$r = \frac{\lambda + n}{e^{At\frac{(1+\alpha)}{\alpha}} - 1}. \tag{1}$$

This applies when the number of initial food items in a patch follows the negative binomial distribution with the mean (μ) and variance (v):

$$\begin{aligned} \mu &= \lambda\alpha \\ v &= \lambda\alpha(1+\alpha) \end{aligned}. \tag{2}$$

We solve Equation (2) for the negative binomial parameters:

$$\begin{aligned} \lambda &= \frac{\mu}{\alpha} \\ \alpha &= \frac{v}{\mu} - 1 \end{aligned}, \tag{3}$$

and substitute for λ in Equation (1),

$$r = \frac{\frac{\mu}{\alpha} + n}{e^{At\frac{(1+\alpha)}{\alpha}} - 1}, \tag{4}$$

and then substitute for α in Equation (4):

$$r = \frac{\frac{\mu}{\frac{v}{\mu}-1}+n}{e^{At}\frac{\frac{v}{\mu}}{\frac{v}{\mu}-1}-1} = \frac{\mu+n\left(\frac{v}{\mu}-1\right)}{e^{At}\frac{v}{\mu}-\frac{v}{\mu}+1}. \tag{5}$$

We then simplify Equation (5) to get

$$r = \frac{\mu-n\left(1-\frac{v}{\mu}\right)}{\left(e^{At}-1\right)\frac{v}{\mu}+1}, \tag{6}$$

which is equivalent to Equation (1). For the binomial distribution the remaining number of prey follows

$$r = \frac{M-n}{e^{At}\frac{(1-q)}{q}+1}. \tag{7}$$

and the mean (μ) and variance (v)

$$\begin{aligned} \mu &= Mq \\ v &= Mq(1-q) \end{aligned}. \tag{8}$$

If Equation (7) and Equation (8) are substituted for M= -λ and q= -α, they become Equation (1) and Equation (2), respectively, and hence leads to Equation (6). For the poisson distribution

$$r = \mu e^{-At}, \tag{9}$$

according to Iwasa *et al*. [1]. If we consider that v=μ for the poisson distribution, Equation (6) simplifies to Equation (9).

Reference List

1. Iwasa Y, Higashi M, Yamamura N (1981) Prey distribution as a factor determining the choice of optimal foraging strategy. Am Nat 117: 710-723.
