# Supplementary material for: A Concept of Bayesian Regulation in Fisheries Management
Source: PLoS One. 2014 Nov 3;9(11):e111614. doi: 10.1371/journal.pone.0111614 (PMC4218784; doi:10.1371/journal.pone.0111614)
Supplement: Appendix S2 — Description of the stochastic operating model of the Baltic Sea herring. (PDF) [file pone.0111614.s002.pdf]

Supporting Information - Appendix S2

The stochastic operating model has numbers-at-age (NAA) and weight-at-age (WAA) as variables for age-class 1 to 8+, in which 8+ is an accumulated age-class of eight-year-olds and older. The dynamic equations for NAA and WAA are:

$$N_{a,y+1} = \begin{cases} R_y, & a = 1 \\ N_{a-1,y} \exp\left(-Z_{a-1,y}\right), & a = 2...7 \\ N_{a-1,y} \exp\left(-Z_{a-1,y}\right) + N_{a,y} \exp\left(-Z_{a-1,y}\right), & a = 8+ \end{cases} \tag{1}$$

$$\overline{W}_{a,y+1} = \begin{cases} \overline{W}_{1,y}, & a = 1 \\ \overline{W}_{a-1,y} + G_{a-1,y}, & a = 2...7 \\ \dfrac{\left(\overline{W}_{a-1,y} + G_{a-1,y}\right) N_{a-1,y} \exp\left(-Z_{a-1,y}\right) + \left(\overline{W}_{a,y} + G_{a,y}\right) N_{a,y} \exp\left(-Z_{a,y}\right)}{N_{a-1,y} \exp\left(-Z_{a-1,y}\right) + N_{a,y} \exp\left(-Z_{a,y}\right)}, & a = 8+ \end{cases} \tag{2}$$

where $N_{a,y}$ are numbers at age a in year y, and $\overline{W}_{a,y}$ is the average weight-at-age on 1 January. R is a recruitment function, Z is a mortality function, G is growth function, and $\overline{W}_{1,y}$ is how recruits are assigned their weight. The mortality,

$$Z_{a,y} = p\left(qf_y^* + m_a + v_a C_y + \varepsilon_y\right), \tag{3}$$

where m_a is the age-specific baseline mortality, and v_a is the parameter for the predation in relation to total cod biomass C_y in year y (cod being the main predator on herring in the Baltic Sea). This and the other functions of the SOM add a stochastic normal distributed error $\varepsilon_{a,y}$. Parameter p is the part of the year, in Equation (3), when the mortality rate

applies. Recruitment is based on the SSB where two- and three year olds have maturity rates of 70 % and 90 % respectively, and older have 100 % maturity with no skipped spawning [1],

$$R_{y+1} = \mathrm{SSB}_y (b + d\mathrm{SSB}_y + \varepsilon_y), \tag{4}$$

where *b* and *d* are estimated parameters. Spawning takes place during the spring and a partial (p=0.3) natural mortality and body growth is used for SSB calculations. Body growth is of the von Bertalanffy type,

$$G_{a,y} = p\left(c + k_y + k_a + l\bar{W}_{a,y} + \varepsilon_{a,y}\right), \tag{5}$$

in which c is a constant, k_y the year-specific growth, k_a the age effect, l the weight-effect parameter. The average weight of recruits is determined by the proportional contribution from parental age-classes and the average weight-at-age of the parent female,

$$\bar{W}_{1,y+1} = k\frac{\sum_{a>1}\left(N^a_{1,y}\bar{W}_{y,a}\right)}{\sum_{a>1} N^a_{1,y}} + \varepsilon_y, \tag{6}$$

where $N^a_{1,y}$ is the number of 1-year-olds from mothers aged a, and k is a proportionality constant. Cod abundance in Equation (3) and year-specific growth in Equation (5) are subject to normally distributed stochastic environmental noise. For more details and parameter values see Holmgren et al. [2].

Reference List

1. ICES (2010) Report of the Baltic Fisheries Assessment Working Group (WGBFAS), 15 - 22 April 2010, ICES Headquarters, Copenhagen. 633 pp. ICES CM 2010/ACOM:10.

2. Holmgren NMA, Norrström N, Aps R, Kuikka S (2012) MSY-orientated management of Baltic Sea herring (Clupea harengus) during different ecosystem regimes. ICES J Mar Sci 69: 257-266.
